# Neurometabolic correlates of posturography in normal aging and older adults with mild cognitive impairment: Evidence from a ^1^H-MRS study

**DOI:** 10.1016/j.nicl.2022.103304

**Published:** 2022-12-24

**Authors:** Oron Levin, Wouter A.J. Vints, Gal Ziv, Gintarė Katkutė, Simona Kušleikienė, Kristina Valatkevičienė, Samrat Sheoran, Margarita Drozdova-Statkevičienė, Rymantė Gleiznienė, Mati Pääsuke, Vilma Dudonienė, Uwe Himmelreich, Vida J. Česnaitienė, Nerijus Masiulis

**Affiliations:** a Department of Health Promotion and Rehabilitation, Lithuanian Sports University, LT-44221 Kaunas, Lithuania; b Movement Control & Neuroplasticity Research Group, Group Biomedical Sciences, KU Leuven, Heverlee 3001, Belgium; c Department of Rehabilitation Medicine Research School CAPHRI, Maastricht University P.O. Box 616, 6200 MD Maastricht, the Netherlands; d The Academic College at Wingate, Netanya 4290200, Israel; e Department of Radiology, Medical Academy, Lithuanian University of Health Sciences, Kaunas, Lithuania; f Faculty of Kinesiology, Sport, and Recreation, University of Alberta, Edmonton, Canada; g Institute of Sport Sciences and Physiotherapy, University of Tartu, Estonia; h Biomedical MRI Unit, Department of Imaging and Pathology, Group Biomedical Sciences, KU Leuven, Leuven 3000, Belgium; i Department of Rehabilitation, Physical and Sports Medicine, Institute of Health Science, Vilnius University, Vilnius, Lithuania; j Centre of Expertise in Rehabilitation and Audiology, Adelante Zorggroep, Hoensbroek, The Netherlands

**Keywords:** Aging, Balance control, Brain neurometabolites, Dual-task effect, Postural stability

## Abstract

Proton magnetic resonance spectroscopy (^1^H-MRS) holds promise for revealing and understanding neurodegenerative processes associated with cognitive and functional impairments in aging. In the present study, we examined the neurometabolic correlates of balance performance in 42 cognitively intact older adults (healthy controls - HC) and 26 older individuals that were diagnosed with mild cognitive impairment (MCI). Neurometabolite ratios of total *N*-acetyl aspartate (tNAA), glutamate-glutamine complex (Glx), total choline (tCho) and myo-inositol (mIns) relative to total creatine (tCr) were assessed using single voxel ^1^H-MRS in four different brain regions. Regions of interest were the left hippocampus (HPC), dorsal posterior cingulate cortex (dPCC), left sensorimotor cortex (SM1), and right dorsolateral prefrontal cortex (dlPFC). Center-of-pressure velocity (Vcop) and dual task effect (DTE) were used as measures of balance performance. Results indicated no significant group differences in neurometabolite ratios and balance performance measures. However, our observations revealed that higher tCho/tCr and mIns/tCr in hippocampus and dPCC were generic predictors of worse balance performance, suggesting that neuroinflammatory processes in these regions might be a driving factor for impaired balance performance in aging. Further, we found that higher tNAA/tCr and mIns/tCr and lower Glx/tCr in left SM1 were predictors of better balance performance in MCI but not in HC. The latter observation hints at the possibility that individuals with MCI may upregulate balance control through recruitment of sensorimotor pathways.

## Highlights

• Neurometabolic correlates of balance were studied in older adults with/without MCI.
• Metabolite ratios to creatine were assessed in 5 voxel locations using ^1^H-MRS.
• Elevated hippocampus NAA and myoinositol predicted higher sway velocity in non-MCI.
• Lower sensorimotor NAA and myoinositol were related to higher sway velocity in MCI.

## 1 Introduction

Balance is fundamental to humans of all ages, but becomes increasingly critical with aging (Winter, 1995). Among the studies showing an effect of age on balance, the obtained results were mainly related to a larger decline in postural stability under multitask conditions (e.g., Granacher et al., 2011, Huxhold et al., 2006; for reviews, see Boisgontier et al., 2013, Petrigna et al., 2021). The negative effect of age on balance is attributed primarily to sensorimotor dysfunctions (Bugnariu and Fung, 2007), muscle weakness (Morse et al., 2004), and structural changes in brain grey and white matter (Boisgontier et al., 2017, Van Impe et al., 2012). However, evidence from multiple independent studies suggests that older adults are able to compensate for declines in postural control by increasing the allocation of attentional resources to the postural task (e.g., Drozdova-Statkevičienė et al., 2018, Doumas et al., 2008, Donker et al., 2007, Stins et al., 2009; for a review see Stins and Beek, 2012). The availability of attentional resources and the ability to allocate attention efficiently is declining with age (Huang et al., 2017, Ward and Frackowiak, 2003, Seidler et al., 2010). As such it is expected that the allocation of attentional resources toward a secondary cognitive task during standing or walking would have a greater interference effect on balance in older adults with impaired cognitive function than in older adults with normal cognitive abilities or young adults (e.g., Gonçalves et al., 2018, Marsh and Geel, 2000, Teasdale and Simoneau, 2001). This could explain why older adults with mild cognitive impairment (MCI) are at a higher risk of developing impaired balance behavior (Agrawal et al., 2020, Chantanachai et al., 2022, Ide et al., 2022, Taylor et al., 2014; for a review see Chantanachai et al., 2021).

Neurodegenerative processes in aging and age-related pathological conditions are typically characterized by alterations in neurometabolite concentrations (e.g., Cleeland et al., 2019, Wang et al., 2015). For example, changes in the regional levels of *N*-acetylaspartate/*N*-acetyl-aspartyl-glutamate complex (tNAA), choline-phosphorylcholine-glycerophosphorylcholine complex (tCho), myo-inositol (mIns), gamma-aminobutyric acid (GABA), and glutamate-glutamine complex (Glx) which can be monitored in-vivo with proton-magnetic resonance spectroscopy (^1^H-MRS), have been identified as potential biomarkers of disease progression in neurodegenerative disorders and dementia (Ben Salem et al., 2008, Ding et al., 2008, Kantarci et al., 2007; Weerasekera et al., 2018; Zanigni et al., 2015). In healthy human volunteers, in-vivo quantification of brain neurometabolites with ^1^H-MRS typically shows age-related declines in regional levels of multiple neurometabolites, including NAA, Glx, and GABA and increases in the levels of Cho and mIns (Ding et al., 2016, Gao et al., 2013, Levin et al., 2019, Porges et al., 2017, Reyngoudt et al., 2012, Vints et al., 2022, Wijtenburg et al., 2013, Zahr et al., 2013). For example, lower concentrations of NAA and Glx in sensorimotor cortex and lower Glx in the striatum were found to be strong predictors of performance declines on a variety of motor and cognitive tests performed by apparently healthy older adults (Levin et al., 2019, Weerasekera et al., 2020, Zahr et al., 2008, Zahr et al., 2013).

Alterations in neurometabolite concentrations or neurometabolite ratios have also been shown to play a pivotal role as mediators of progressive performance declines observed in older adults with MCI or patients with dementia-related disorders (e.g., Kantarci et al., 2002, Lyros et al., 2020, Mitolo et al., 2019, Oeltzschner et al., 2019). Specifically, low tNAA/tCr and elevated mIns/tCr and tCho/tCr in posterior cingulate cortex and/or hippocampus were reported as early predictors of transition from MCI to Alzheimer’s disease (Liu et al., 2021, Zhao et al., 2021). Finally, elevated levels of mIns in hippocampus and high levels of tCho and glutamate in the anterior cingulate cortex were found to be associated with worse performance on a working memory task as well as elevated low-grade systemic inflammation (Lind et al., 2021, Vints et al., 2022). Therefore, examining associations between postural stability and neurometabolite levels in key brain regions involved in balance control can shed light on processes that may lead to balance instability in older adults with MCI who are at high risk for falls and fall-related injuries (Liu-Ambrose et al., 2008). However, the above-mentioned evidence leaves us with a knowledge gap about the neurometabolic correlates of balance control in older adults, in general, and older individuals with MCI in particular.

In the current study, we aim to answer the following questions: (1) to what extent can neurometabolic changes related to MCI predict balance performance, and (2) is there a difference in the association between neurometabolites and balance between healthy participants and participants with MCI? We focused specifically on possible associations between balance performance and expressions of tNAA/tCr tCho/tCr and mIns/tCr in hippocampus, hippocampal gyrus, posterior cingulate cortex, sensorimotor cortex, and prefrontal cortex. We hypothesized that (1) lower tNAA/tCr and elevated tCho/tCr and mIns/tCr, which are considered as biomarkers of neurodegeneration and neuroinflammation, respectively (e.g., Lind et al., 2020, Liu et al., 2021, Vints et al., 2022, Zhao et al., 2021), would be related to reduced balance performance in general. Moreover, we hypothesized that (2) specific neurometabolic markers of neurodegeneration (tNAA/tCr) and neuroinflammation (mIns/tCr and tCho/tCr) in the hippocampus would predict worse balance performance of older individuals with MCI more than normative older adults. The latter hypothesis is supported by a number of observations indicating that accelerated neurodegenerative processes in the hippocampus interrupt vestibular functioning, specifically in individuals with MCI (Jacob et al., 2020, Micarelli et al., 2017, Micarelli et al., 2018, Semenov et al., 2016, Wang et al., 2022, Wei et al., 2018, Wei et al., 2019). We further examined the neurometabolic correlates of balance control in relation to measures of tNAA/tCr, tCho/tCr, mIns/tCr, and Glx/tCr in prefrontal, posterior cingulate, and sensorimotor regions due to their expected involvement in attentional control of balance (Petrigna et al., 2021, Sugihara et al., 2021).

## 2 Materials and methods

### 2.1 Participants

Participants were 68 (37 females and 31 males), apparently healthy, older adults aged 60–85 years that were recruited from the same pool of participants as in Vints et al (2022). The experimental protocol was approved by the local Medical Ethics Committee for Biomedical Research (No. BE-10–7), and a written informed consent was obtained from all participants prior to their inclusion in the study. Individuals with diagnosed central nervous system (CNS) injuries, alcohol abuse, diabetes, musculoskeletal disorders, neurodegenerative diseases, or any oncological diseases were excluded from the study. All participants were asked to report age, sex, smoking status, and educational level (i.e., basic education, secondary education or higher education) and were instructed not to exercise prior to all appointments and cognitive tests. Body mass index (BMI) and percent body fat (% BF) were estimated by using leg-to-leg bio-impedance analysis (BIA, Tanita TBF-300-A). All participants underwent the Montreal Cognitive Assessment (MoCA) test and psychological health assessment tests that were conducted in a clinical environment by a qualified mental health-care specialist (co-author SK). Clinical diagnosis for MCI was made according to the International Classification of Diseases (ICD-10) and Petersen criteria (Petersen et al., 2014); yielding two groups: 42 (24 females) healthy controls (HC) [Age: 67.3 ± 5.1 years; MoCA: 26.8 ± 1.9] and 26 (13 females) MCIs [Age: 72.7 ± 6.8 years; MoCA 22.4 ± 2.0]. None of the participants were diagnosed with dementia and all participants with MCI had a score of 19 or above on the MoCA test. Exploratory analysis with independent samples t-tests revealed that men with MCI were older than their HC counterparts (p < 0.001) whereas no group difference in age was found between women (p = 0.075). For both genders, individuals with MCI were characterized by a lower weight, lower % BF, and lower BMI. However, differences were marginal or did not reach significance after applying the Bonferroni correction for multiple comparisons (all p ≥ 0.032); for details, see Supplementary Table A.1.

### 2.2 Experimental measurements (posturography)

**Fig. 1:**
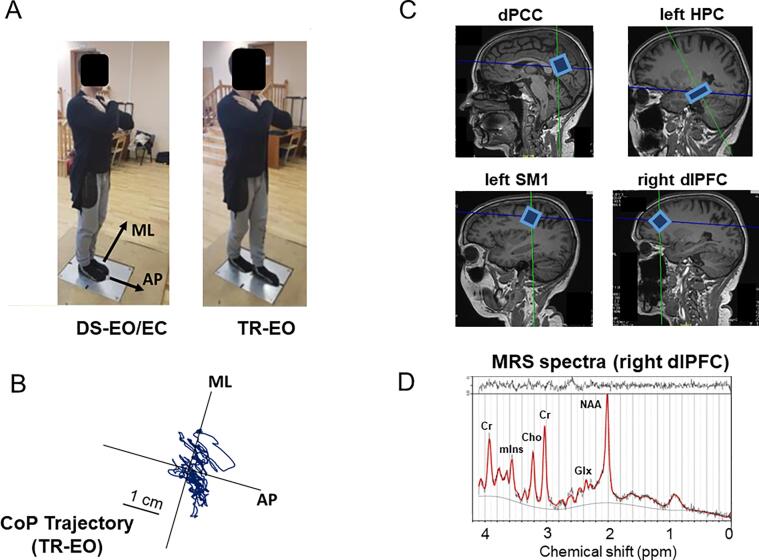
**(A-B)** Illustration of stance conditions and representative CoP trajectory. **(C)** Voxel placement in the dorsal posterior cingulate cortex (dPCC), left hippocampus (HPC), left primary sensorimotor cortex (SM1), and right dorsolateral prefrontal cortex (dlPFC). **(D)** Representative spectra (black) and LCModel fits (red) for PRESS data from right dlPFC. (For interpretation of the references to colour in this figure legend, the reader is referred to the web version of this article.)

A posturography method with a single piezoelectric force plate (KISTLER, Switzerland, Slimline System 9286) was used to measure postural sway activity. The signals collected from the force plate were digitized at 100 Hz and were stored on PC for an off-line analysis. The application point (center of pressure - CoP) of the measured foot–ground reaction forces in the anteroposterior (AP) and mediolateral (ML) directions was calculated based on the known geometric locations of the piezoelectric transducers. Participants were tested barefoot in three stance conditions: wide double-stance with eyes open (DS-EO), wide double-stance with eyes closed (DS-EC), and Tandem Romberg stance with eyes open (TR-EO), as previously described (Drozdova-Statkevičienė et al., 2018; see Fig. 1A-B). For the TR-EO stance condition, the participants were instructed to step on the force plate, to place their feet in a heel-to-toe position along the midline of the platform and to stand still in this stance position with eyes open. The positioning of the feet was determined in the familiarization trial to allow maximum conformability of the participant. The selected foot positions remained the same for all the testing trials. The CoP recordings were repeated under single and dual task conditions, yielding six experimental conditions, that were presented in a counterbalanced order and were repeated 3 times (with approximately 1 min between two consecutive trials). For the dual task conditions, we used a Mathematical Counting task similar to that used by Drozdova-Statkevičienė et al., 2018, Drozdova-Statkevičienė et al., 2021. Negative or positive one-digit integer-numbers (10 in total) were presented vocally in each trial at 2 s intervals and participants were instructed to calculate and remember the sum. Participants were instructed to concentrate on the calculation and to memorize the calculated sum in their mind at each step throughout the trial. At the end of each trial, participants were asked to verbally report the correct answer (e.g., the correct answer “10” was expected for the numbers [+6, +8, −3, +9, −5, −1, +6, −8, −4, +2]). Data collection lasted 25 s of which the last 20 s were processed. Mean and linear trends of the AP and ML components of the CoP trajectory were subtracted and a fourth-order low pass Butterworth bi-directional filter with a cutoff frequency of 15 Hz was applied on the raw CoP displacement vectors. CoP velocity vector (Vcop) were calculated from the displacement AP and ML vectors of the CoP, using a custom-written MATLAB script (MathWorks, Natick, MA). AP and ML Vcop data in each of the six experimental conditions were averaged over the three repeated trials. Finally, the dual task effect (DTE) was quantified as the percent change of sway activity (as measured with Vcop) from dual-to single-task relative to its single task measure. Positive DTE values indicate deterioration of balance stability (i.e., an increase of Vcop from single to dual task) whereas negative DTE values indicate improvement of balance stability (i.e., a decrease of Vcop from single to dual task).

### 2.3 Brain imaging and ^1^H-MRS

Brain scanning consisted of anatomical whole brain MRI and single voxel ^1^H-MRS at the five voxel locations with a total length of about 90 min per participant as previously described by Vints et al. (2022). All scanning sessions were conducted using a Siemens 3 T Skyra Magnetic Resonance scanner (Siemens Healthineers, Erlangen, Germany) with a 32-channel receiver head coil. A high-resolution T1-weighted structural MR image (repetition time (TR) = 2200 ms, echo time (TE) = 2.48 ms, 0.9 × 0.9 × 1.0 mm^3^ voxels, field of view: 230 × 256 mm, number of sagittal slices = 176) was used to acquire a 3D magnetization prepared gradient echo (MPRAGE) image. ^1^H-MR spectra were acquired in five voxel locations, namely the dorsal posterior cingulate cortex (dPCC), left primary sensorimotor cortex (left SM1), left hippocampus (left HPC), left middle temporal cortex (left MTC), and right dorsolateral prefrontal cortex (right dlPFC) (see Fig. 1C; left MTC not shown). MRS data were acquired using a point resolved spectroscopy (PRESS) sequence (TR = 2000 ms, TE = 30 ms, number of averages = 128, spectral bandwidth = 2000 Hz, data size = 1024 points) with chemical shift selective (CHESS) water suppression (bandwidth = 50 Hz). The voxel sizes were: (i) 1.6 × 1.6 × 1.6 cm^3^ in the dorsal PCC, left SM1 and right DLPFC voxels, (ii) 20 × 12 × 16 cm^3^ in the left MTC, (iii) 26 × 12 × 12 in the left HPC. The unsuppressed water signal was also acquired for absolute metabolite quantification using the same acquisition parameters. Voxel-specific shimming was performed using automated B0-field mapping followed by manual adjustment to reduce the water signal full width at half maximum (FWHM) below 15 Hz. In total 340 spectra (=68 participants × 5 regions) were collected. Spectra were visually checked to ensure the absence of artefacts prior to quantification with LCModel (version 6.3.1-R). The basis set for LCModel consisted of 27 basis spectra including: alanine (Ala), aspartate (Asp), creatine (Cr), phosphocreatine (PCr), γ-aminobutyric acid (GABA), glucose (Glc), glutamine (Gln), glutamate (Glu), glycerophosphocholine (GPC), phosphorylcholine (PCh), myoinositol (mIns), lactate (Lac), *N*-acetyl aspartate (NAA), *N*-acetyl-aspartyl-glutamate (NAAG), scyllo-inositol (Scyllo), taurine (Tau), negative creatine methylene (-CrCH2), guanidinoacetate (Gua), lipids [Lip09, Lip13a, Lip13b, and Lip20] and macromolecules [MM09, MM12, MM14, MM17 and MM20]. Major metabolite complexes were: (1) total NAA (tNAA) composed of NAA and NAAG, (2) total creatine (tCr) composed of Cr and PCr, (3) total choline (tCho) composed of GPC and PCh, (4) mIns, and (5) the glutamate–glutamine complex (Glx) (Fig. 1D); see Zöllner et al (2021). Quantified neurometabolites were the ratios to tCr of tNAA, tCho, mIns, and Glx (i.e., tNAA/tCr, tCho/tCr, mIns/tCr, and Glx/tCr). Spectra with FHWM > 15 Hz, Cramér-Rao lower bound (CRLB) > 20 % and/or signal-to-noise ratio (SNR) < 5 were not included in the statistical analyses. This resulted in the elimination of 17 spectra (8.10 %) from the HC group and 15 (11.5 %) spectra from the MCI group. Due to a large amount of missing data from the left MTC (22.1 %), this region was not included in the analyses. Variables of interest were the concentration ratios of tNAA/tCr, Glx/tCr, tCho/tCr, and mIns/tCr in four regions of interest (i.e., dPCC, left SM1, left HPC and right dlPFC), yielding 16 neurometabolic measures. All included spectra had SNR ≥ 6, CRLB ≤ 19 % and FWHM ≤ 0.105 ppm. A detailed description of MRS acquisition protocol, MRS data management and MRS data quality were added in Appendix B of the Supplementary Materials in line with the MRSinMRS Reporting Checklist (Lin et al., 2021).

### 2.4 Statistical analysis

#### 2.4.1 Group differences

All statistical analyses were performed using the open-source statistical software JASP (version 0.16.2; JASP Team, 2022). Group differences in posturography (sway velocity and dual task effect) were analyzed using a set of ANCOVAs with age, gender, MoCA, height, weight, and % BF as covariates and Group as a between-subject factor. A three-way [Group (HC/MCI) × Vision (EO/EC) × Task (Single/Dual)] ANCOVA with repeated measures on Vision and Task factors was applied to examine differences in sway velocity under dual-task conditions in double-stance (DS) with and without visual feedback. A two-way Group × Task ANCOVA with repeated measures on Task factor was applied to examine group-differences in sway velocity under dual-task conditions in the TR-EO stance conditions. A two-way Group × Vision ANCOVA with repeated measures on the Vision factor was applied to examine group-differences in DTE in the DS stance condition and one-way ANCOVA was applied to examine group-differences in DTE in the TR-EO stance condition. Finally, group differences in local neurometabolite ratios (tNAA/tCr, tCho/tCr, mIns/tCr and Glx/tCr) within each of the four regions were examined using a set of two-way [Group (HC/MCI) × Region (dPCC/left HPC/left SM1/right dlPFC)] ANCOVAs with repeated measures on the Region factor and age, gender, and % BF as covariates. The assumption of sphericity was tested using the Mauchly’s sphericity test and the Greenhouse–Geisser correction was applied when this assumption was not met. Whenever appropriate, post-hoc analyses were conducted using the Tukey’s test (in the case of significant main effects or interactions) or the Bonferroni’s correction (in the case of exploratory analysis). Results of the ANCOVAs can be found in Supplementary Tables A.2 - A.3 (sway activity - Vcop), Supplementary Table A.4 (dual-task effect - DTE), and Supplementary Table A.5 (neurometabolite ratios: tNAA/tCr, tCho/tCr, mIns/tCr, and Glx/tCr).

#### 2.4.2 Bivariate correlation and exploratory regression analyses

The associations between tNAA/tCr, tCho/tCr, mIns/tCr and Glx/tCr ratios in the four regions and posturographic measures of sway velocity (Vcop) and DTE were examined using Pearson’s correlations. The correlations were repeated for each of the HC and MCI groups separately. P values were corrected for multiple comparisons using the false discovery rate (FDR) approach (Benjamini and Hochberg, 1995) with p = 0.05 as a limit. Correlations were considered as statistically significant if surviving FDR corrections for 16 multiple comparisons (=4 Neurometabolite ratios × 4 Regions) at p < 0.05. Non-significant results with a moderate effect size (r > 0.30) were reported as trends if p-values were below 0.10. A Fisher r-to-z transformation was used for comparison between correlations in order to identify group differences in neurometabolic correlates of posturography between HC and MCI. Results are summarized in Supplementary Tables A.6 - A.8 (Vcop) and Supplementary Tables A.9 - A.10 (DTE). Finally, stepwise multiple linear regression models were employed to determine the unique variance contributed by specific neurometabolites to the performance of the balance task. Neurometabolites were removed from the regression model in a subsequent stepwise step, using the default threshold of p = 0.05 for entry and p = 0.10 for removal of a variable into the model. The stepwise selection process was repeated with no a priori selection of included metabolites. Specifically, entering all four neurometabolite ratios (i.e., tNAA/tCr, tCho/tCr, mIns/tCr, and Glx/tCr) in the four regions of interest (i.e., dPCC, left HPC, left SM1, and right dlPFC) as potential independent variables in order to ensure that the methodological choice described above did not influence the results (e.g., Levin et al., 2019). Analyses were performed for the full (combined) sample of HCs and MCIs (n = 52) who had a complete dataset of posturography and MRS measures.

## 3 Results

### 3.1 Group differences

#### 3.1.1 Sway activity

**Fig. 2:**
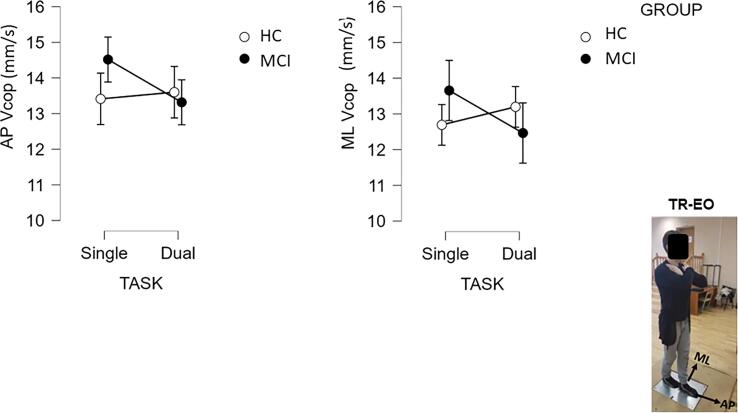
Group differenced in sway velocity as function of task (single-task verses dual-task) during Tandem Romberg stance with eyes open. AP = Anteroposterior, ML = Medio-lateral, Vcop = Center of Pressure velocity.

One HC participant (man, 64 years old) and one MCI participant (woman, 73 years old) did not complete the DS-EC task. Data from two MCI participants (both men, 77 and 81 years old) were discarded because of excessive AP and/or ML sway (Vcop > 50 mm/s) in the DS-EC or TR-EO stance conditions. All remaining posturography measures were found to be within the excepted range of AP (Vcop ≤ 39.0 mm/s) and ML (Vcop ≤ 32.2 mm/s) sway velocity in similar age cohorts (Roman-Liu, 2018). For illustrations, see Figure A.1 in the Supplementary Materials. The results of the three-way Group X Stance X Task ANCOVA on sway in the DS-EO and DS-EC stance conditions (Table A.2 in the Supplementary Materials) revealed no significant main effects or interactions [AP Vcop: all F(1,55) ≤ 2.10, p > 0.10, η_p_^2^ < 0.04; ML Vcop: all F(1,55) ≤ 0.87, p > 0.30, η_p_^2^ < 0.02]. In contrast, findings from the two-way Group X Task ANCOVA on sway activity in the TR-EO stance yielded a significant Group X Task interactions for both AP sway [AP Vcop: F(1,56) = 5.27, p = 0.026, η_p_^2^ = 0.09] and ML sway [ML Vcop: F(1,56) = 4.26p = 0.044, η_p_^2^ = 0.07]. Post hoc analyses (Tukey tests) revealed that individuals with MCI displayed lower AP and ML Vcop in the dual task as compared with Vcop observed in the single task condition (Fig. 2). Nonetheless, decreased sway from single- to dual-task in the MCI group was not statistically significant (p ≥ 0.104). No such trend was found for the HC (p > 0.40). Overall, the current findings suggest that individuals with MCI tended to increase postural stability when facing a challenging balance task.

#### 3.1.2 Dual task effect

No significant main effects or interactions were found for DTE scores in the DS-EO and DS-EC stance conditions [DTE for AP sway: all F(1,56) ≤ 2.09, p > 0.1, η_p_^2^ < 0.04; DTE for ML sway: all F(1,56) ≤ 1.08, p > 0.30, η_p_^2^ ≤ 0.02]; see Table A.4 and Figure A.2 in the Supplementary Materials. These observations were consistent with the results from corresponding analyses on the measures of sway activity under the same stance conditions. A significant main effect of Group was found for AP and ML DTE scores in the TR-EO stance [DTE for AP sway: F(1,56) = 4.58, p = 0.037, η_p_^2^ = 0.08; DTE for ML sway: F(1,56) = 4.22, p = 0.045, η_p_^2^ = 0.07]. Inspection of the marginal means showed a consistent decrease of sway from single to dual task in MCIs, resulting in negative mean DTE scores [AP sway: −13.16 % (95 % CI: −25.92, −0.40); ML sway: −11.31 % (95 % CI: −24.98, +2.35)]. In contrast to MCIs, individuals in the HC group tended to increase sway, resulting in positive DTE [AP sway: 5.86 % (95 % CI: −2.97, +14.70); ML sway: 8.24 % (95 % CI: −1.22, +17.71)].

#### 3.1.3 Neurometabolite ratios

The results of all ANCOVAs showed no significant group effects or interactions between group and region for all tested neurometabolite ratios [for all ANCOVAs, Group main effect and Group X Region interaction: F ≤ 1.38, p > 0.2, η_p_^2^ < 0.03]. Overall, these observations suggest that no group differences in neurometabolite ratios between HC and MCI exist; for illustrations see Table A.5 and Figure A.3 in the Supplementary Materials.

### 3.2 Associations between neurometabolite ratios and posturography measures

#### 3.2.1 Neurometabolite ratios and sway activity

##### 3.2.1.1 Dual stance

Overall, our findings suggest that associations between neurometabolite ratios and sway in the DS-EO and DS-EC stance conditions were more frequent in the HC group and, to a lesser extent, in MCIs (Table 1). For HC, significant associations were observed between left HPC mIns/tCr and sway velocity measures in AP or ML directions (uncorrected p ≤ 0.002; all survived FDR correction). In addition, for the DS-EC stance, we observed a significant positive association between left HPC tNAA/tCr ratio and magnitudes of AP and ML sway velocity in dual-task (uncorrected p ≤ 0.003; all survived FDR correction). For MCI, all associations or trends between neurometabolite ratios and posturography measures of sway activity in the DS-EO and DS-EC stance conditions did not survive FDR correction. However, associations or trends with a moderate effect size were found primarily between left SM1 tNAA/tCr and the magnitude of AP sway velocity for both single-and dual-task; all were negative associations. Similar trends of positive associations between neurometabolite ratios and sway velocity were found for ML sway velocity and left SM1 Glx/tCr for the DS-EO stance and for ML sway velocity and left HPC tCho/tCr for the DS-EC stance; both in single task conditions. Significant group differences in correlations were found between: (1) Left HPC tNAA/tCr and AP sway velocity during DS-EO for single-task [r_HC_ = 0.312, r_MCI_ = -0.175, |z| = 1.66, p = 0.049]. (2) Left HPC tNAA/tCr and AP sway velocity during DS-EC stance for single-task [r_HC_ = 0.353, r_MCI_ = −0.200, |z| = 1.89, p = 0.029] and dual-task [r_HC_ = 0.534, r_MCI_ = −0.112, |z| = 2.35, p = 0.011]. (3) Left SM1 Glx/Cr and magnitude of ML sway velocity for single-task [r_HC_ = -0.192, r_MCI_ = 0.440, |z| = 2.38, p = 0.009] and dual-task [r_HC_ = −0.106, r_MCI_ = 0.363, |z| = 1.74, p = 0.041] (Table 1); for the full correlation results see Supplementary Tables A.6 – A.7.

**Table 1:** Main neurometabolic correlates of sway activity (Vcop) for normal older adults (HC) and older adults with MCI in dual-stance with eyes open and eyes closed.

	Group (Ns/Nd)	Single Task		Dual task	
		AP Vcop	ML Vcop	AP Vcop	ML Vcop
**DS-EO**					
l. HPC	HC	0.312†	0.306†	0.325*	0.209
tNAA/tCr	MCI	−0.175	0.223	−0.084	0.199
	|z|	1.66*	< 1	1.40	< 1
l. HPC	HC	0.496**	0.338*	0.497**	0.380*
mIns/tCr	MCI	0.122	0.331	0.288	0.187
	|z|	1.40	< 1	< 1	< 1
l. SM1	HC	−0.179	0.008	−0.103	−0.113
tNAA/tCr	MCI	−0.422*	−0.180	−0.417*	−0.159
	|z|	<1	<1	1.22	<1
l. SM1	HC	0.089	−0.192	0.235	−0.106
Glx/tCr	MCI	0.159	0.440*	0.203	0.363†
	|z|	<1	2.38**	<1	1.74*
**DS-EC**					
l. HPC	HC	0.353*	0.236	0.534**	0.476**
tNAA/tCr	MCI	−0.200	0.204	−0.112	0.313
	|z|	1.89*	<1	2.35*	<1
l. HPC	HC	0.211	0.174	0.326*	0.262
tCho/tCr	MCI	0.279	0.485*	0.170	0.333
	|z|	<1	1.17	<1	<1
l. HPC	HC	0.440**	0.378*	0.560***	0.424**
mIns/tCr	MCI	0.120	0.188	0.058	0.152
	|z|	1.17	<1	1.86*	<1
l. SM1	HC	−0.159	−0.141	−0.081	−0.026
tNAA/tCr	MCI	−0.458*	−0.237	−0.384†	−0.181
	|z|	1.21	<1	1.17	<1

Significant group differences in correlation coefficients (Fisher r-to-z transformation) are highlighted in **bold**. For the full data set, see: Supplementary Tables A.6 and A.7.

**Abbreviations:** AP = Anteroposterior, ML = Medio-lateral, Vcop = Centre of pressure mean velocity, DS-EO = dual-stance with eyes open, DS-EC = dual-stance with eyes closed, dPCC = dorsal posterior cingulate cortex, l. HPC = left hippocampus, l. SM1 = left primary sensorimotor cortex, r. dlPFC = right dorsolateral prefrontal cortex, tNAA = total *N*-acetyl aspartate, tCho = total choline, mIns = myoinositol, tCr = total creatine, Glx = glutamate-glutamine complex. * p < 0.05, ** p < 0.01, **†** p < 0.1 with a moderate effect side (| r | > 0.3); all uncorrected p-values.

##### 3.2.1.2 TR-EO stance

No significant correlations between neurometabolite ratios and Vcop measures were found after FDR corrections. Associations or trends between neurometabolite ratios and posturography in the TR-EO stance conditions (Table 2) were observed only for MCI and were characterized primarily by negative associations between left SM1 NAA/tCr and mIns/tCr ratios and the magnitude of AP sway velocity. Significant group differences in correlations between neurometabolite ratios and posturography measures of sway in the TR-EO stance were found between: Left SM1 tNAA/tCr ratio and AP Vcop at single-task [rHC = −0.077, r_MCI_ = −0.522, z = 1.817, p = 0.035] and dual-task [rHC = 0.042, r_MCI_ = −0.496, z = 2.121, p = 0.017]. (2) left SM1 mIns/tCr and AP Vcop in single-task [r_HC_ = -0.048, r_MCI_ = -0.556, z = 2.096, p = 0.018] and dual-task [r_HC_ = 0.061, r_MCI_ = -0.569, z = 2.560, p = 0.005]. In addition, we observed a significant group difference for left SM1 mIns/tCr and ML Vcop during dual-task [r_HC_ = 0.191, r_MCI_ = -0.391, z = 2.23, p = 0.013] (Table 2); for the full correlation results see Supplementary Table A.8.

**Table 2:** Main neurometabolic correlates of sway activity (Vcop) for normal older adults (HC) and older adults with MCI in Tandem Romberg stance.

	Group	Single Task		Dual Task	
		AP Vcop	ML Vcop	AP Vcop	ML Vcop
**TR-EO**					
dPCC	HC	0.288	0.264	0.154	0.176
tCho/tCr	MCI	0.333	0.260	0.372†	0.381†
	|z|	< 1	< 1	< 1	< 1
l. SM1	HC	−0.077	0.107	0.042	0.157
tNAA/tCr	MCI	−0.522*	−0.262	−0.496*	−0.143
	|z|	1.82*	1.36	2.12*	1.09
l. SM1	HC	−0.048	0.168	0.061	0.199
mIns/tCr	MCI	−0.556**	−0.276	−0.569**	−0.391†
	|z|	2.10*	1.64	2.56**	2.23*
r. dlPFC	HC	0.175	0.228	0.090	0.173
tCho/tCr	MCI	0.226	0.342	0.157	0.441*
	|z|	<1	<1	<1	<1

Significant group differences in correlation coefficients (Fisher r-to-z transformation) are highlighted in **bold**. For the full data set, see: Supplementary Table A.8.

**Abbreviations:** AP = Anteroposterior, ML = Medio-lateral, Vcop = Centre of pressure mean velocity, TR-EO = Tandem Romberg stance with eyes open, dPCC = dorsal posterior cingulate cortex, l. HPC = left hippocampus, l. SM1 = left primary sensorimotor cortex, r. dlPFC = right dorsolateral prefrontal cortex, tNAA = total *N*-acetyl aspartate, tCho = total choline, mIns = myoinositol, tCr = total creatine. * p < 0.05, ** p < 0.01, **†** p < 0.1 with a moderate effect side (|r| > 0.3); all uncorrected p-values.

#### 3.2.2 Neurometabolite ratios and dual task effect

##### 3.2.2.1 Dual stance

No significant correlations between neurometabolite ratios and DTE scores were found after FDR corrections. Associations with a moderate effect size were observed primarily in MCI (Table 3). For HC, a positive association with a moderate effect size was found between right dlPFC tNAA/tCr and DTE score for ML sway in the DS-EO stance. For the MCI group, positive associations with moderate effect sizes were found between dPCC tNAA/tCr and left HPC mIns/tCr and AP DTE scores and a negative association with a moderate effect size was found between dPCC mIns/tCr and ML DTE scores; all during DS-EO. Significant group differences were found for correlations between AP DTE and dPCC tNAA/tCr [r_HC_ = −0.207, r_MCI_ = 0.453, |z| = 2.49, p = 0.006] and AP DTE and left HPC mIns/Cr [r_HC_ = 0.013, r_MCI_ = 0.475, z = −1.67, p = 0.048] (Table 3); for the full correlation results see Supplementary Table A.9.

**Table 3:** Main neurometabolic correlates of dual-task effect (DTE) for normal older adults (HC) and older adults with MCI.

	Group (Ns/Nd)	DS-EO		TR-EO	
		AP DTE	ML DTE	AP DTE	ML DTE
dPCC	HC(41/41)	−0.207	−0.203		
tNAA/tCr	MCI (22/21)	0.453*	−0.289		
	|z|	2.49**	< 1		
dPCC	HC (41/41)	−0.265	−0.215		
mIns/tCr	MCI (22/21)	−0.182	−0.453*		
	|z|	<1	<1		
l. HPC	HC			0.144	0.153
tNAA/tCr	MCI			0.457*	0.212
	|z|			1.15	<1
l. HPC	HC (38/38)	0.013	0.175		
mIns/tCr	MCI (19/18)	0.475*	−0.354		
	|z|	1.67*	1.77*		
l. SM1	HC (41/41)	−0.017	−0.153		
tCho/tCr	MCI (23/22)	−0.414*	−0.125		
	|z|	1.53	<1		
r. dlPFC	HC (41)	−0.080	−0.325*		
tNAA/tCr	MCI (22/21)	−0.054	−0.250		
	|z|	<1	<1		
r. dlPFC				0.231	0.260
tNAA/tCr				0.094	0.390**†**
				<1	<1

Significant group differences in correlation coefficients (Fisher r-to-z transformation) are highlighted in **bold**. For the full data set, see: Supplementary Tables A.9 and A.10. No significant correlations with DTE were observed for dual-stance with eyes closed (DS-EC).

**Abbreviations:** AP = Anteroposterior, ML = Medio-lateral, DS-EO = dual-stance with eyes open, TR-EO = Tandem Romberg stance with eyes open. dPCC = dorsal posterior cingulate cortex, l. HPC = left hippocampus, SM1 = left primary sensorimotor cortex, r. dlPFC = right dorsolateral prefrontal cortex, tNAA = total *N*-acetyl aspartate, tCho = total choline, mIns = myoinositol, tCr = total creatine. *p < 0.05, ** p < 0.01, **†** p < 0.1 with a moderate effect side (|r| > 0.3); all uncorrected p-values.

##### 3.2.2.2 TR-EO stance

Positive associations with moderate effect sizes were observed for MCI between AP DTE scores and tNAA/tCr in the left HPC and right dlPFC (Table 3). However, group differences were not significant (|z| ≤ 1.15, p > 0.1); for the full correlation table see Supplementary Table A.10.

### 3.3 Exploratory regression analyses from the full sample:

#### 3.3.1 Sway activity

The regression model revealed that magnitude of sway in the DS-EO and DS-EC stance conditions were associated primarily with left HPC mIns/tCr ratio (Table 4). Specifically: (1) AP Vcop during DS-EO dual-task (R^2^ = 0.163, p = 0.003) and DS-EC single-task (R^2^ = 0.160, p = 0.003); (2) ML Vcop during DS-EO single-task (R^2^ = 0.109, p = 0.016) and dual-task (R^2^ = 0.108, p = 0.016); (3) ML Vcop during DS-EC single-task (R^2^ = 0.126, p = 0.009). For the remaining conditions: dPCC tCho/tCr together with left HPC mIns/tCr explained 24.4 % of the variance of AP Vcop during DS-EO single-task (p ≤ 0.019) and left HPC tNAA/tCr together with left HPC mIns/tCr explained 25.2 % of the variance of ML Vcop during DS-EC dual-task (p ≤ 0.040). Finally, left HPC tNAA/tCr was the only predictor of ML Vcop during DS-EC dual-task (R^2^ = 0.193, p = 0.001). For the TR-EO stance, tCho/tCr ratio in the dPCC was found to be the only predictor of AP Vcop at dual-task (R^2^ = 0.173, p = 0.002) and the only predictor of ML Vcop at both single-task (R^2^ = 0.083, p = 0.037) and dual-task (R^2^ = 0.099, p ≤ 0.022). Finally, tCho/tCr and Glx/tCr ratios in the dPCC and tCho/tCr ratio in the left SM1 collectively predicted 36.9 % of the variance of AP Vcop during single task (p ≤ 0.046) with dPCC tCho/tCr alone explaining 20.2 % of the variance (p < 0.001).

**Table 4:** Results of the multiple regression models between the posturographic measures of sway velocity (Vcop) and their neurometabolite predictors^1^.

			R^2^		β	t	p-value
AP Vcop	DS-EO	ST	0.244	dPCC tCho/tCr	0.335	2.669	0.010
				l. HPC mIns/tCr	0.304	2.246	0.019
		DT	0.163	l. HPC mIns/tCr	0.403	3.147	0.003
	DS-EC	ST	0.160	l. HPC mIns/tCr	0.400	3.120	0.003
		DT	0.252	l. HPC mIns/tCr	0.309	2.270	0.028
				l. HPC tNAA/tCr	0.287	2.106	0.040
	TR-EO	ST	0.369	dPCC tCho/tCr	0.642	5.136	< 0.001
				l. SM1 tCho/tCr	−0.367	−2.966	0.005
				dPCC Glx/tCr	−0.237	−2.052	0.046
		DT	0.173	dPCC tCho/tCr	0.416	3.264	0.002
ML Vcop	DS-EO	ST	0.109	l. HPC mIns/tCr	0.300	2.498	0.016
		DT	0.108	l. HPC mIns/tCr	0.328	2.483	0.016
	DS-EC	ST	0.126	l. HPC mIns/tCr	0.355	2.713	0.009
		DT	0.193	l. HPC tNAA/tCr	0.439	3.453	0.001
	TR-EO	ST	0.083	dPCC tCho/tCr	0.288	2.147	0.037
		DT	0.099	dPCC tCho/tCr	0.314	2.365	0.022

Analyses were performed for the full (combined) sample of HCs and MCIs (n = 52). R^2^ = multiple R^2^, β = standardized regression coefficient.

**Abbreviations:** AP = Anteroposterior, ML = Medio-lateral, Vcop = CoP velocity, DS-EO = dual-stance with eyes open, DS-EC = dual-stance with eyes closed, TR-EO = Tandem Romberg stance with eyes open, ST = single task, DT = dual task. dPCC = dorsal posterior cingulate cortex, l. HPC = left hippocampus, l. SM1 = left primary sensorimotor cortex, tNAA = total *N*-acetyl aspartate, tCho = total choline, mIns = myo-inositol, tCr = total creatine, Glx = glutamate-glutamine complex.

#### 3.3.2 Dual-task effect

Right dlPFC tNAA/tCr and dPCC mIns/tCr together explained 22.8 % of the variance of DTE in the ML direction during DS-EO and dPCC tNAA/tCr was the only predictor of DTE in the ML direction during TR-EO (R^2^ = 0.076, p = 0.045). None of the neurometabolite candidates that have been entered to our regression model were found to be significant predictors of the variance of AP DTE scores (Table 5).

**Table 5:** Results of the multiple regression models between the posturographic measures of dual-task effect (DTE) and their neurometabolite predictors.

		R^2^		β	t	p-value
ML DTE	DS-EO	0.228	r. dlPFC tNAA/tCr	−0.378	−3.037	0.004
			dPCC mIns/tCr	−0.306	−2.462	0.017
	TR-EO	0.076	r. dlPFC tNAA/tCr	0.276	2.054	0.045

Analyses were performed for the full (combined) sample of HCs and MCIs (n = 52). R^2^ = multiple R^2^, β = standardized regression coefficient. No significant regression models were found for AP DTE (for all stance conditions) and ML DTE for the DS-EC stance condition.

**Abbreviations:** AP = Anteroposterior, ML = Medio-lateral, Vcop = CoP velocity, DS-EO = dual-stance with eyes open, DS-EC = dual-stance with eyes closed, TR-EO = Tandem Romberg stance with eyes open, dPCC = dorsal posterior cingulate cortex, r. dlPFC dorsolateral prefrontal cortex, tNAA = total *N*-acetyl aspartate, mIns = myo-inositol, tCr = total creatine,

## 4 Discussion

### 4.1 General findings

In this exploratory study, we examined the associations between balance performance and neurometabolic biomarkers of cognitive impairments. Our first major observation was that elevated mIns/tCr and tCho/tCr in the posterior cingulate cortex and hippocampus together and independently were predictors of lower balance stability (i.e., high sway velocity); supporting our first hypothesis. This finding provides evidence that age-related effects on balance performance could be explained in part by neuroinflammatory processes such as gliosis. The abovementioned finding is further substantiated by observations from other ^1^H-MRS studies showing associations between cognitive aging and higher expressions of mIns and tCho in hippocampus and cingulate brain areas (Graff-Radford et al., 2014, Lind et al., 2020, Lind et al., 2021, Vints et al., 2022). A second major observation was that MCI and HC showed different patterns of associations between neurometabolite ratios and balance performance measures, thus partly supporting our second hypothesis. Specifically, we found that balance performance in MCI was predicted primarily by tNAA/tCr and mIns/tCr in the sensorimotor cortex but not in the hippocampus, whereas for HC balance performance was predicted primarily by tNAA/tCr and mIns/tCr in the hippocampus. These findings suggest that individuals with MCI may rely more on sensorimotor pathways to regulate balance stability possibly as compensation for the decreased integrity of hippocampal and prefrontal pathways of balance control (Ward and Frackowiak, 2003, Wittenberg et al., 2017).

### 4.2 Group comparison of neurometabolites

The analyses of ^1^H-MRS spectra from dPCC, left HPC, left SM1, and right dlPFC collected in our study revealed no statistical differences between MCI and HC in all neurometabolite measures. The observations are in line with findings from some ^1^H-MRS studies (Lyros et al., 2020, Mitolo et al., 2019), but contradictory to other studies where group differences in concentrations of tNAA, tCho, mIns, Glx, and/or their ratios to tCr were reported (e.g., Kantarci et al., 2007, Oeltzschner et al., 2019, Olson et al., 2008, Targosz-Gajniak et al., 2013, Zhao et al., 2021; for *meta*-analysis see Liu et al., 2021). The absence of significant group differences in tNAA/tCr, mIns/tCr and tCho/tCr between HC and MCI observed in the present study could therefore suggest that individuals who were included in our sample of MCI were at very early stage of neurodegeneration or at low risk of developing dementia (Kantarci, 2013, Kantarci et al., 2002, Kantarci et al., 2007, Metastasio et al., 2006, Zhang et al., 2015). However, it is also possible that absence of a significant group effect in our study is related to the low sample size and high heterogeneity of our cohort. For example, in the study of Olson et al (2008) 47 individuals with MCI were tested which, based on neurometabolic changes, were subdivided into ‘‘atypical’’ (atMCI) and ‘‘typical’’ (tMCI) subgroups. The atMCI subgroup (36 %) was characterized by increased levels of NAA, Cr, and Glx whereas the tMCI (64 %) subgroup were characterized by decreased levels of NAA, Cr, and Glx from baseline to follow-up within one year test–retest interval. Based on their findings, Olson et al (2008) suggested that neurometabolic features may be heterogeneous within the MCI population. This heterogeneity could be the reason for absence of significant group differences between MCI and HC as observed in the present study.

Decreased levels of tNAA and increased levels of tCho and mIns can underlie important structural and physiological changes in the brain that are related to cognitive aging. These neurometabolic differences are thought to reflect age-related neuronal density decrease and demyelination (tNAA), increased glial cell activity (mIns), and membrane alterations (tCho) (e.g., Ding et al., 2016, Eylers et al., 2016, Lind et al., 2021, Vints et al., 2022, Waragai et al., 2017; for a review see Cleeland et al., 2019). Elevated tCho and mIns in the aging brain appears to be a potential marker of brain inflammation, demyelination, gliosis and cognitive decline (Harris et al., 2014, Langer et al., 2021, Lind et al., 2021, Vints et al., 2022). Lower tNAA/tCr and elevated mIns/tCr in hippocampus and posterior cingulate cortex structures of individuals with MCI have been considered as potential biomarkers for high risk of progressing from MCI to Alzheimer’s disease (AD) (Liu et al., 2021). However, results from the same *meta*-analysis suggest that differences between MCI-converter and MCI-stable patients were not statistically significant.

### 4.3 Neurometabolite predictors of balance performance in normal aging and MCI

In contrast to some previous findings (Deschamps et al., 2014, Shin et al., 2011, Yoon et al., 2020) but in line with observations from other studies (e.g., Mignardot et al., 2014, Sparto et al., 2020; for a review see Bahureksa et al., 2017), we found no significant group differences in magnitude of sway velocity or DTE between MCI and HC. This observation is not unexpected in view that MCI is considered as an intermediate stage between the cognitive changes of normal aging and dementia (Petersen, 2000). Balance impairments in MCI often occur in parallel with lower functional status (Ansai et al., 2019, Gonçalves et al., 2018, Yoon et al., 2020), reduced sensorimotor functioning (Taylor et al., 2012), impaired executive control (Delbaere et al., 2012, Taylor et al., 2014, Taylor et al., 2017) and/or poorer performances in cognitive visuospatial domains (Taylor et al., 2014) more than with declines in global cognition or memory (e.g., Ansai et al., 2019, Taylor et al., 2014); for a review see Chantanachai et al (2021). Furthermore, observations from several studies suggests that the prevalence of balance impairments in individuals with MCI may not be consistent across populations and could be defined by other factors such as age, lifestyle, and features of MCI subtypes (e.g., Chantanachai et al., 2022, Tangen et al., 2014, Yoon et al., 2020). Given this inconsistency, we propose that participants in the MCI group in this study were less prone to balance impairments. However, we did not examine specific features of balance behavior within subgroups of MCIs (e.g., amnestic versus non-amnestic) due to insufficient sample sizes. An alternative explanation for the lack of group differences in balance performance is that individuals with MCI may recruit adjacent or alternative pathways to the functionally impaired pathways as a compensatory mechanism (Parra et al., 2013). This assumption is supported in part by the moderate bivariate correlation of sway velocity with tNAA/tCr ratios in left SM1 and right dlPFC, which was observed only in our MCI group.

Our findings showed that, under a cognitive dual-task, individuals with MCI decreased their sway velocity. Similar trends were reported by Swan et al (2007) for a group of healthy young adults demonstrating that difficult cognitive tasks produced a significant decrease in sway that was not seen with easy cognitive tasks. No significant changes in magnitude of sway velocity (neither increase nor decrease) from single to dual task were found in HC. We propose that when facing complex or challenging task conditions, individuals with MCI may activate intrinsic or automatic mechanisms of balance control (more than their HC counterparts) to mitigate the effects of secondary (cognitive) tasks on their balance behavior. This explanation can be further supported by physiological findings showing that individuals with MCI consistently are subjected to a greater cognitive workload as compared to healthy older adults when performing the same task (Ranchet et al., 2017, Gonçalves et al., 2018). The interdependence between balance performance and cognition in older adults, which has been demonstrated to be enhanced in MCI, is characterized by low performance in executive function and working memory (Ide et al., 2022, Montero-Odasso et al., 2009). The evidence for the interplay between poor executive functioning and the magnitude of dual task effect suggest that postural adjustments during dual task may be dependent on the integrity of prefrontal pathways involved in attentional control (e.g., Marusic et al., 2019). Indeed, we found that the magnitude of the dual-task effect observed in our full sample was associated with lower tNAA/tCr ratios in dPCC and dlPFC. The fact that lower tNAA/tCr ratios in the right dlPFC and left SM1 have been found to be associated with higher sway activity in MCI but not in HC suggest that older individuals with MCI may have recruited prefrontal and sensorimotor pathways to reinforce balance control. Finally, a positive association between left SM1 Glx/tCr and the magnitude of sway was observed in MCI. The latter observation hints at the possibility that older adults with MCI may upregulate the activation of pathways responsible for balance control through the increase of glutamatergic signaling.

The findings of the current study are added to the available body of knowledge, highlighting the associations between changes in prefrontal, striatal, or sensorimotor concentrations of tNAA and declines in motor or cognitive functioning in older adults without overt diseases (Ben Salem et al., 2008, Nikolaidis et al., 2017, Levin et al., 2019, Weerasekera et al., 2020, Zahr et al., 2008, Zahr et al., 2013). Reductions of NAA concentrations and lower NAA/Cr ratios are considered as hallmarks of brain atrophy in MCI (Kantarci et al., 2007, Targosz-Gajniak et al., 2013, Zhao et al., 2021); for a *meta*-analysis see Liu et al (2021). Decreased regional levels of NAA are generally considered to be biomarkers of white matter (WM) microstructural declines and demyelination (Wijtenburg et al., 2013, Grossman et al., 2015). The neurodegenerative processes leading to balance impairment in older adults with MCI may, nonetheless, differ substantially from those of older adults without MCI. More specifically, we found that high sway activity in the MCI group was associated primarily with reduced tNAA/tCr in the left SM1. In contrast, high sway activity in HC was associated with elevated mIns/tCr in the hippocampus. Importantly, when applying multiple regression models that included the full sample of HC and MCI, we found that high hippocampus mIns/tCr and dPCC tCho/tCr emerged as principal (and often single) predictors of a large sway velocity in both the AP and ML directions. A significant association between elevated hippocampal mIns/tCr and high levels of sway was observed only for the sample of HC. Elevated ratios of tCho/tCr and mIns/tCr (and increased brain levels of tCho and mIns in general) are considered to be robust biomarkers of neuroinflammatory processes and gliosis that are often seen in older individuals with low-grade systemic inflammation (Lind et al., 2021, Vints et al., 2022). Taking this observation into account, we propose that neuroinflammatory processes in hippocampus and dPCC may be key players in the mechanism(s) underlying postural instability in aging.

The specific impact of cognitive aging on balance performance could nevertheless differ according to the severity of cognitive impairment (Allan et al., 2009, Chantanachai et al., 2022). The absence of differences in signs of neuroinflammation between MCI and HC in our study could suggest that age-related pro-inflammatory processes at the early stages of MCI may be comparable to those underlying normal aging. A longitudinal experimental design involving older adults with normal cognitive functions, individuals with different MCI subtypes is therefore warranted. A possible avenue for future research is to examine the extent by which the observed associations between brain neurometabolites and balance behavior are influenced by the severity and/or subtypes of MCI (e.g., Kantarci et al., 2007, Tumati et al., 2018, Taylor et al., 2017).

## 5 Conclusions

Taken as a whole, findings from the present study indicated that decreased tNAA/tCr, and increased tCho/tCr and mIns/tCr were associated with worse balance performance. More specifically, our observations revealed that deficient regulation of balance control in both normal cognitive aging and MCI might be related, in part, to neuroinflammatory processes in hippocampus and posterior cingulate cortex. This observation adds to a growing body of evidence indicating that neuroinflammation appears to play a crucial role in cognitive and motor functional declines in aging (Lind et al., 2021, Vints et al., 2022). Results from our study also yielded that older adults with MCI seem to rely more on sensorimotor pathways to support postural control under demanding balance conditions. Our findings point at potentially relevant neurometabolic biomarkers for risk assessment of balance instability in cognitive aging and MCI.

## Declaration of Competing Interest

The authors declare that they have no known competing financial interests or personal relationships that could have appeared to influence the work reported in this paper.

## Data Availability

Data will be made available on request.
