# Supplementary material for: Neurometabolic correlates of posturography in normal aging and older adults with mild cognitive impairment: Evidence from a 1H-MRS study
Source: Neuroimage Clin. 2022 Dec 24;37:103304. doi: 10.1016/j.nicl.2022.103304 (PMC9827054; doi:10.1016/j.nicl.2022.103304)
Supplement: Supplementary data 1 [file mmc1.docx]

**Supplementary materials**

**Neurometabolite correlates of posturography** **in normal aging and older adults** **with mild cognitive impairment: evidence from ^1^H-MRS study**

**Appendix A: Supplementary Tables and Figures**

**Table A.1.** Participant demographic characteristics.

|  | Gender | HC  (n=42) | MCI  (n=26) | All  (n=68) | p-value  (t-test)^b^ |
| --- | --- | --- | --- | --- | --- |
| Age (y) | Male | 68.3 (5.2) | 75.3 (4.9) | 71.1 (6.1) | **< 0.001** |
|  | Female | 66.6 (5.0) | 70.4 (7.6) | 68.0 (6.2) | 0.075 |
| MoCA | Male | 26.7 (2.2) | 22.2 (2.0) | 24.9 (3.1) | **< 0.001** |
|  | Female | 26.8 (1.7) | 22.6 (2.1) | 25.2 (2.8) | **< 0.001** |
| ^a^ Height (cm) | Male | 175.5 (4.3) | 172.3 (4.7) | 174.2 (4.7) | 0.062 |
|  | Female | 160.9 (4.9) | 161.9 (7.3) | 161.2 (5.7) | 0.575 |
| ^a^ Weight (kg) | Male | 91.4 (19.5) | 78.3 (11.5) | 86.1 (17.8) | 0.046 |
|  | Female | 73.6 (11.8) | 66.8 (9.8) | 71.2 (11.5) | 0.084 |
| ^a^ % BF | Male | 28.2 (8.1) | 22.0 (7.6) | 25.7 (8.3) | 0.043 |
|  | Female | 37.6 (6.6) | 31.9 (8.7) | 35.6 (7.8) | 0.032 |
| ^a^ BMI (kg/m^2^) | Male | 29.6 (5.9) | 26.4 (3.7) | 28.3 (5.3) | 0.099 |
|  | Female | 28.5 (4.7) | 25.5 (3.3) | 27.4 (4.5) | 0.046 |
| Participants | Male | 18 (26.5%) | 12 (17.6%) |  |  |
|  | Female | 24 (35.3 %) | 14 (20.6%) |  |  |

HC = healthy controls, MCI = mild cognitive impairment, MoCA = Montreal Cognitive Assessment test, % BF = percent body fat, BMI = Body Mass Index.

^a^ Missing data from one HC female. Continuous parameters are expressed as mean values (SD); categorical parameters are expressed as n (% of total).

^b^ Uncorrected p-values. Significant group differences (Bonferroni correction) are shown in **bold**.

**Table A.2.** Results of the 3-way ANCOVAs for sway velocity (Vcop) during DS-EO and DS-EC stance.

|  | **df** | **Vcop AP** | | **Vcop ML** | |
| --- | --- | --- | --- | --- | --- |
|  |  | **F** | **η²_p_** | **F** | **η²_p_** |
| Condition (EO/EC) | 1,56 | 0.00 | 0.000 | 0.47 | 0.008 |
| Task (Single/Dual) | 1,56 | 0.03 | 0.000 | 0.02 | 0.000 |
| Condition × Task | 1,56 | 0.30 | 0.005 | 0.68 | 0.012 |
| Group (HC/MCI) | 1,56 | 0.53 | 0.009 | 0.61 | 0.011 |
| Group × Condition | 1,56 | 0.39 | 0.007 | 0.31 | 0.005 |
| Group × Task | 1,56 | 0.35 | 0.006 | 0.02 | 0.000 |
| Group × Condition × Task | 1,56 | 0.12 | 0.002 | 0.36 | 0.006 |

df = degrees of freedom. η²_p_ = eta squared. * p < 0.05; ** p < 0.01;

AP = Anteroposterior; ML= Mediolateral

**Table A.3.** Results of the 2-ways ANCOVAs for sway velocity (Vcop) during TR-EO stance.

|  | **df** | **Vcop AP** | | **Vcop ML** | |
| --- | --- | --- | --- | --- | --- |
| AP Vcop |  | **F** | **η²_p_** | **F** | **η²_p_** |
| Task (Single/dual) | 1,56 | 1.50 | 0.026 | 2.83 | 0.048 |
| Group (HC/MCI) | 1,56 | 0.67 | 0.012 | 2.39 | 0.041 |
| Group × Task | 1,56 | 5.27* | 0.086 | 4.26* | 0.071 |

df = degrees of freedom. η²_p_ = eta squared. * p < 0.05; ** p < 0.01;.

AP = Anteroposterior; ML= Mediolateral

**Table A.4.** Results of the 2-way ANCOVAs for dual task effect (DTE) during dual stance with eyes open (DS-EO) and eyes closed (DS-EC).

|  | **df** | **DTE AP** | | **DTE ML** | |
| --- | --- | --- | --- | --- | --- |
| AP Vcop |  | **F** | **η²_p_** | **F** | **η²_p_** |
| Condition (EO/EC) | 1,56 | 0.65 | 0.011 | 0.79 | 0.014 |
| Group (HC/MCI) | 1,56 | 1.92 | 0.033 | 0.88 | 0.015 |
| Group × Condition | 1,56 | 2.09 | 0.036 | 1.08 | 0.019 |

df = degrees of freedom. η²_p_ = eta squared. * p < 0.05; ** p < 0.01;

AP = Anteroposterior; ML= Mediolateral

**Table A.5.** Results of the 2-way ANCOVAs for neurometabolite ratios with Regions (dPCC, l..HPC, l.SM1, and r.dlPFC) as within subject factors.

| tNAA/tCr | df | **F** | **η²_p_** |
| --- | --- | --- | --- |
| Region | 2.632 | 1.97 | 0.038 |
| Group (HC/MCI) | 1,50 | 0.72 | 0.014 |
| Group × Region | 2.632 | 0.11 | 0.002 |

| tCho/tCr | df | **F** | **η²_p_** |
| --- | --- | --- | --- |
| Region | 2.630 | 2.87* | 0.055 |
| Group (HC/MCI) | 1,49 | 1.38 | 0.027 |
| Group × Region | 2.630 | 0.31 | 0.006 |

| mIns/tCr | df | **F** | **η²_p_** |
| --- | --- | --- | --- |
| Region | 2.144 | 0.15 | 0.003 |
| Group (HC/MCI) | 1,49 | 0.22 | 0.033 |
| Group × Region | 2.144 | 0.15 | 0.003 |

| Glx/tCr | df | **F** | **η²_p_** |
| --- | --- | --- | --- |
| Region | 2.618 | 0.16 | 0.003 |
| Group (HC/MCI) | 1,49 | 0.39 | 0.008 |
| Group × Region | 2.618 | 0.37 | 0.007 |

df = degrees of freedom. η²_p_ = eta squared. * p < 0.05; ** p < 0.01;

**Table A.6.** Neurometabolic correlates of sway activity during double-stance with eyes open (DS-EO).

|  | Group  (ns/nd) | Single Task | | Dual task | |
| --- | --- | --- | --- | --- | --- |
|  |  | AP Vcop | ML Vcop | AP Vcop | ML Vcop |
| dPCC | HC (41/41) | 0.180 | 0.248 | 0.044 | 0.039 |
| tNAA/tCr | MCI (22/22) | 0.096 | 0.230 | 0.273 | 0.092 |
|  |  |  |  |  |  |
| dPCC | HC (41/41) | 0.206 | 0.196 | 0.102 | 0.073 |
| tCho/tCr | MCI (22/22) | 0.306 | 0.141 | 0.252 | 0.035 |
|  |  |  |  |  |  |
| dPCC | HC (40/41) | 0.141 | 0.325 | 0.009 | 0.084 |
| mIns/tCr | MCI (22/22) | 0.341 | 0.205 | 0.237 | 0.003 |
|  |  |  |  |  |  |
| dPCC | HC (40/41) | -0.101 | -0.181 | -0.118 | -0.276 |
| Glx/tCr | MCI (22/22) | 0.145 | 0.253 | 0.248 | 0.118 |
|  |  |  |  |  |  |
| l. HPC | HC (38/38) | 0.312† | 0.306† | 0.325* | 0.209 |
| tNAA/tCr | MCI (19/19) | -0.175 | 0.223 | -0.084 | 0.199 |
|  |  |  |  |  |  |
| l. HPC | HC (38/38) | 0.281 | 0.230 | 0.237 | 0.151 |
| tCho/tCr | MCI (19/19) | 0.232 | 0.292 | 0.286 | 0.341 |
|  |  |  |  |  |  |
| l. HPC | HC (38/38) | 0.496** | 0.388* | 0.497** | 0.380* |
| mIns/tCr | MCI (19/19) | 0.122 | 0.331 | 0.288 | 0.187 |
|  |  |  |  |  |  |
| l. HPC | HC (38/38) | 0.156 | 0.190 | 0.037 | 0.035 |
| Glx/tCr | MCI (19/19) | -0.086 | 0.136 | 0.104 | 0.184 |
|  |  |  |  |  |  |
| l. SM1 | HC (41/41) | -0.179 | 0.008 | -0.103 | -0.113 |
| tNAA/tCr | MCI (23/23) | -0.422* | -0.180 | -0.417* | -0.159 |
|  |  |  |  |  |  |
| l. SM1 | HC (41/41) | 0.013 | 0.100 | 0.009 | -0.021 |
| tCho/tCr | MCI (23/23) | -0.230 | -0.007 | -0.363† | -0.038 |
|  |  |  |  |  |  |
| l. SM1 | HC (41/41) | 0.076 | 0.084 | 0.144 | 0.191 |
| mIns/tCr | MCI (23/23) | -0.188 | -0.056 | -0.239 | -0.172 |
|  |  |  |  |  |  |
| l. SM1 | HC (41/41) | 0.089 | -0.192 | 0.235 | -0.106 |
| Glx/tCr | MCI (23/23) | 0.159 | 0.440* | 0.203 | 0.363† |
|  |  |  |  |  |  |
| r. dlPFC | HC (41/41) | -0.250 | -0.070 | -0.278 | -0.295 |
| tNAA/tCr | MCI (22/22) | -0.121 | 0.232 | -0.144 | 0.110 |
|  |  |  |  |  |  |
| r. dlPFC | HC (41/41) | 0.083 | 0.126 | 0.072 | 0.043 |
| tCho/tCr | MCI (22/22) | 0.037 | 0.156 | 0.051 | 0.222 |
|  |  |  |  |  |  |
| r. dlPFC | HC (41/41) | 0.130 | 0.056 | 0.096 | 0.003 |
| mIns/tCr | MCI (22/22) | 0.100 | 0.208 | -0.049 | 0.122 |
|  |  |  |  |  |  |
| r. dlPFC | HC (41/41) | -0.181 | -0.240 | -0.174 | -0.235 |
| Glx/tCr | MCI (22/22) | 0.113 | 0.234 | 0.086 | 0.207 |
|  |  |  |  |  |  |

AP = Anteroposterior, ML = Medio-lateral, Vcop = Centre of pressure mean velocity, dPCC = dorsal posterior cingulate cortex, l. HPC = left hippocampus, l. SM1 = left sensorimotor cortex, r. dlPFC = right dorsolateral prefrontal cortex, tNAA = total N-acetyl aspartate, tCho = total choline, mIns = myoinositol, Glx = glutamate//glutamine complex, tCr = total creatine. * p < 0.05, ** p < 0.01, **†** p < 0.1 with a moderate effect side (|r| > 0.3). Meaningful associations highlighted in **bold**.

**Table A.7.** Neurometabolic correlates of sway activity for HC and MCI during double-stance with eyes closed (DS-EC).

|  | Group  (ns/nd) | Single Task | | Dual task | |
| --- | --- | --- | --- | --- | --- |
|  |  | AP Vcop | ML Vcop | AP Vcop | ML Vcop |
| dPCC | HC (41/41) | 0.118 | 0.074 | 0.089 | 0.059 |
| tNAA/tCr | MCI (22/22) | 0.016 | 0.284 | -0.148 | 0.076 |
|  |  |  |  |  |  |
| dPCC | HC (41/41) | 0.091 | 0.086 | 0.114 | 0.088 |
| tCho/tCr | MCI (22/22) | 0.232 | 0.171 | 0.292 | 0.166 |
|  |  |  |  |  |  |
| dPCC | HC (41/41) | 0.080 | 0.134 | 0.080 | 0.213 |
| mIns/tCr | MCI (22/22) | 0.162 | 0.023 | 0.075 | -0.096 |
|  |  |  |  |  |  |
| dPCC | HC (41/41) | -0.062 | -0.208 | -0.039 | -0.234 |
| Glx/tCr | MCI (22/22) | 0.083 | 0.045 | 0.147 | 0.085 |
|  |  |  |  |  |  |
| l. HPC | HC (38/38) | 0.353* | 0.236 | 0.534*** | 0.476** |
| tNAA/tCr | MCI (19/19) | -0.200 | 0.204 | -0.112 | 0.313 |
|  |  |  |  |  |  |
| l. HPC | HC (38/38) | 0.211 | 0.174 | 0.326* | 0.262 |
| tCho/tCr | MCI (19/19) | 0.279 | 0.485* | 0.170 | 0.333 |
|  |  |  |  |  |  |
| l. HPC | HC (38/38) | 0.440** | 0.378* | 0.560*** | 0.424** |
| mIns/tCr | MCI (19/19) | 0.120 | 0.188 | 0.058 | 0.152 |
|  |  |  |  |  |  |
| l. HPC | HC (38/38) | 0.013 | -0.069 | 0.137 | 0.100 |
| Glx/tCr | MCI (19/19) | -0.043 | 0.067 | -0.057 | 0.187 |
|  |  |  |  |  |  |
| l. SM1 | HC (41/41) | -0.159 | -0.141 | -0.081 | -0.026 |
| tNAA/tCr | MCI (23/23) | **-0.458*** | -0.237 | -0.384† | -0.181 |
|  |  |  |  |  |  |
| l. SM1 | HC (41/41) | -0.135 | -0.100 | -0.063 | -0.010 |
| tCho/tCr | MCI (23/23) | -0.153 | 0.082 | 0.082 | 0.235 |
|  |  |  |  |  |  |
| l. SM1 | HC (41/41) | -0.026 | 0.040 | 0.004 | 0.111 |
| mIns/tCr | MCI (23/23) | -0.246 | -0.234 | -0.193 | -0.164 |
|  |  |  |  |  |  |
| l. SM1 | HC (41/41) | 0.285 | -0.032 | 0.302† | 0.004 |
| Glx/tCr | MCI (23/23) | 0.111 | 0.199 | 0.067 | 0.216 |
|  |  |  |  |  |  |
| r. dlPFC | HC (41/41) | -0.279 | -0.255 | -0.207 | -0.163 |
| tNAA/tCr | MCI (22/22) | -0.137 | 0.213 | -0.066 | 0.169 |
|  |  |  |  |  |  |
| r. dlPFC | HC (41/41) | 0.067 | 0.100 | 0.088 | 0.142 |
| tCho/tCr | MCI (22/22) | 0.052 | 0.225 | 0.330 | 0.371 |
|  |  |  |  |  |  |
| r. dlPFC | HC (41/41) | 0.170 | 0.152 | 0.147 | 0.069 |
| mIns/tCr | MCI (22/22) | 0.247 | 0.194 | 0.197 | 0.150 |
|  |  |  |  |  |  |
| r. dlPFC | HC (41/41) | -0.127 | -0.233 | -0.028 | -0.150 |
| Glx/tCr | MCI (22/22) | 0.218 | 0.234 | 0.042 | 0.139 |
|  |  |  |  |  |  |

AP = Anteroposterior, ML = Medio-lateral, Vcop = Centre of pressure mean velocity, dPCC = dorsal posterior cingulate cortex, l. HPC = left hippocampus, l. SM1 = left sensorimotor cortex, r. dlPFC = right dorsolateral prefrontal cortex, tNAA = total N-acetyl aspartate, tCho = total choline, mIns = myoinositol, Glx = glutamate//glutamine complex, tCr = total creatine. * p < 0.05, ** p < 0.01, **†** p < 0.1 with a moderate effect side (|r| > 0.3). ns = number of samples at single task, nd = number of samples at dual task.

**Table A.8.** Neurometabolic correlates of sway activity for HC and MCI during Tandem Romberg stance with eyes open (TR-EO).

|  | Group  (ns/nd) | Single Task | | Dual task | |
| --- | --- | --- | --- | --- | --- |
|  |  | AP Vcop | ML Vcop | AP Vcop | ML Vcop |
| dPCC | HC (40/40) | 0.015 | 0.018 | -0.032 | 0.091 |
| tNAA/tCr | MCI (22/22) | 0.010 | 0.087 | 0.069 | 0.165 |
|  |  |  |  |  |  |
| dPCC | HC (40/40) | 0.288 | 0.264 | 0.154 | 0.176 |
| tCho/tCr | MCI (22/22) | 0.333 | 0.260 | 0.372† | 0.381† |
|  |  |  |  |  |  |
| dPCC | HC (40/40) | -0.019 | 0.106 | -0.065 | 0.073 |
| mIns/tCr | MCI (22/22) | -0.060 | 0.078 | -0.147 | -0.188 |
|  |  |  |  |  |  |
| dPCC | HC (40/40) | -0.044 | -0.163 | 0.064 | -0.042 |
| Glx/tCr | MCI (22/22) | -0.068 | 0.122 | -0.159 | -0.021 |
|  |  |  |  |  |  |
| l. HPC | HC (37/37) | 0.163 | -0.041 | 0.175 | 0.022 |
| tNAA/tCr | MCI (19/19) | 0.074 | 0.275 | 0.155 | 0.351 |
|  |  |  |  |  |  |
| l. HPC | HC (37/37) | 0.274 | 0.128 | 0.206 | 0.067 |
| tCho/tCr | MCI (19/19) | 0.278 | 0.125 | 0.349 | 0.252 |
|  |  |  |  |  |  |
| l. HPC | HC (37/37) | 0.272 | -0.117 | 0.177 | -0.119 |
| mIns/tCr | MCI (19/19) | -0.140 | 0.063 | -0.132 | -0.069 |
|  |  |  |  |  |  |
| l. HPC | HC (37/37) | 0.157 | 0.113 | 0.276 | 0.147 |
| Glx/tCr | MCI (19/19) | 0.081 | 0.053 | 0.078 | 0.032 |
|  |  |  |  |  |  |
| l. SM1 | HC (41/41) | -0.077 | 0.107 | 0.042 | 0.157 |
| tNAA/tCr | MCI (23/23) | -0.522* | -0.262 | -0.496* | -0.143 |
|  |  |  |  |  |  |
| l. SM1 | HC (41/41) | -0.046 | 0.108 | 0.053 | 0.139 |
| tCho/tCr | MCI (23/23) | -0.038 | 0.136 | -0.067 | 0.168 |
|  | \| |  |  |  |  |
| l. SM1 | HC (41/41) | -0.048 | 0.168 | 0.061 | 0.199 |
| mIns/tCr | MCI (23/23) | -0.556** | -0.276 | -0.569** | -0.391† |
|  |  |  |  |  |  |
| l. SM1 | HC (41/41) | 0.084 | 0.009 | 0.088 | -0.033 |
| Glx/tCr | MCI (23/23) | -0.111 | 0.177 | -0.094 | 0.066 |
|  |  |  |  |  |  |
| r. dlPFC | HC (40/40) | -0.188 | -0.091 | -0.087 | 0.042 |
| tNAA/tCr | MCI (22/22) | 0.089 | 0.123 | 0.122 | 0.349 |
|  |  |  |  |  |  |
| r. dlPFC | HC (40/40) | 0.175 | 0.228 | 0.090 | 0.173 |
| tCho/tCr | MCI (22/22) | 0.226 | 0.342 | 0.157 | 0.441* |
|  |  |  |  |  |  |
| r. dlPFC | HC (40/40) | -0.033 | -0.013 | -0.012 | 0.016 |
| mIns/tCr | MCI (22/22) | -0.066 | -0.084 | -0.065 | -0.171 |
|  |  |  |  |  |  |
| r. dlPFC | HC (40/40) | -0.072 | -0.187 | -0.038 | -0.189 |
| Glx/tCr | MCI (22/22) | 0.107 | -0.085 | 0.101 | -0.172 |
|  |  |  |  |  |  |

AP = Anteroposterior, ML = Medio-lateral, Vcop = Centre of pressure mean velocity, dPCC = dorsal posterior cingulate cortex, l. HPC = left hippocampus, l. SM1 = left sensorimotor cortex, r. dlPFC = right dorsolateral prefrontal cortex, tNAA = total N-acetyl aspartate, tCho = total choline, mIns = myoinositol, Glx = glutamate//glutamine complex, tCr = total creatine. * p < 0.05, ** p < 0.01, **†** p < 0.1 with a moderate effect side (|r| > 0.3). ns = number of samples at single task, nd = number of samples at dual task.

**Table A.9.** Neurometabolic correlates of dual task effect (DTE) for HC and MCI during double stance with eyes open (DS-EO) and eyes closed (DS-EC).

|  | Group  (no/nc) | DS-EO | | DS-EC | |
| --- | --- | --- | --- | --- | --- |
|  |  | AP DTE | ML DTE | AP DTE | ML DTE |
| dPCC | HC (41/41) | -0.207 | -0.203 | -0.155 | -0.057 |
| tNAA/tCr | MCI (22/22) | 0.453* | -0.289 | -0.226 | -0.340 |
|  |  |  |  |  |  |
| dPCC | HC (41/41) | -0.160 | -0.150 | -0.082 | -0.112 |
| tCho/tCr | MCI (22/22) | -0.070 | -0.311 | -0.066 | -0.103 |
|  |  |  |  |  |  |
| dPCC | HC (41/41) | -0.265 | -0.215 | -0.129 | 0.007 |
| mIns/tCr | MCI (22/22) | -0.182 | -0.453* | -0.213 | -0.237 |
|  |  |  |  |  |  |
| dPCC | HC (41/41) | -0.077 | -0.189 | 0.012 | -0.024 |
| Glx/tCr | MCI (22/22) | 0.255 | -0.221 | 0.080 | 0.066 |
|  |  |  |  |  |  |
| l. HPC | HC (37/38) | 0.028 | -0.031 | 0.193 | 0.297 |
| tNAA/tCr | MCI (19/19) | 0.123 | -0.031 | 0.153 | 0.092 |
|  |  |  |  |  |  |
| l. HPC | HC (37/38) | -0.083 | -0.097 | 0.092 | 0.031 |
| tCho/tCr | MCI (19/19) | 0.226 | 0.112 | -0.313 | -0.375 |
|  |  |  |  |  |  |
| l. HPC | HC (37/38) | 0.013 | 0.175 | 0.045 | 0.008 |
| mIns/tCr | MCI (19/19) | 0.457* | -0.354 | -0.215 | -0.094 |
|  |  |  |  |  |  |
| l. HPC | HC (37/38) | -0.147 | -0.180 | 0.101 | 0.155 |
| Glx/tCr | MCI (19/19) | 0.386 | 0.056 | 0.203 | 0.280 |
|  |  |  |  |  |  |
| l. SM1 | HC (41/41) | 0.039 | -0.168 | 0.206 | 0.142 |
| tNAA/tCr | MCI (23/23) | -0.118 | 0.019 | 0.247 | 0.022 |
|  |  |  |  |  |  |
| l. SM1 | HC (41/41) | -0.017 | -0.153 | 0.165 | 0.131 |
| tCho/tCr | MCI (23/23) | -0.414* | -0.125 | 0.337 | 0.128 |
|  |  |  |  |  |  |
| l. SM1 | HC (41/41) | 0.113 | 0.189 | 0.100 | 0.154 |
| mIns/tCr | MCI (23/23) | -0.160 | -0.260 | 0.111 | 0.039 |
|  |  |  |  |  |  |
| l. SM1 | HC (41/41) | 0.186 | 0.076 | 0.048 | 0.076 |
| Glx/tCr | MCI (23/23) | 0.162 | -0.145 | -0.122 | -0.004 |
|  |  |  |  |  |  |
| r. dlPFC | HC (40/41) | -0.080 | -0.325* | 0.283 | 0.092 |
| tNAA/tCr | MCI (22/22) | -0.054 | -0.250 | 0.013 | -0.034 |
|  |  |  |  |  |  |
| r. dlPFC | HC (40/41) | -0.006 | -0.073 | 0.062 | 0.074 |
| tCho/tCr | MCI (22/22) | 0.013 | 0.070 | 0.286 | 0.191 |
|  |  |  |  |  |  |
| r. dlPFC | HC (40/41) | -0.074 | -0.045 | -0.068 | -0.095 |
| mIns/tCr | MCI (22/22) | -0.276 | -0.272 | -0.232 | -0.105 |
|  |  |  |  |  |  |
| r. dlPFC | HC (40/41) | -0.037 | -0.105 | 0.043 | 0.021 |
| Glx/tCr | MCI (22/22) | 0.058 | -0.104 | -0.284 | -0.162 |
|  |  |  |  |  |  |

AP = Anteroposterior, ML = Medio-lateral, Vcop = Centre of pressure mean velocity, dPCC = dorsal posterior cingulate cortex, l. HPC = left hippocampus, l. SM1 = left sensorimotor cortex, r. dlPFC = right dorsolateral prefrontal cortex, tNAA = total N-acetyl aspartate, tCho = total choline, mIns = myoinositol, Glx = glutamate//glutamine complex, tCr = total creatine. * p < 0.05, ** p < 0.01, **†** p < 0.1 with a moderate effect side (|r| > 0.3). no = number of samples at eyes open, nc = number of samples at eyes closed.

**Table A.10.** Neurometabolic correlates of dual-task effect (DTE) for HC and MCI during Tandem Romberg stance with eyes open (TR-EO).

|  | Group (N) | DTE | |
| --- | --- | --- | --- |
|  |  | AP | ML |
| dPCC | HC (40) | -0.018 | -0.234 |
| tNAA/tCr | MCI (22) | 0.273 | 0.210 |
|  |  |  |  |
| dPCC | HC (40) | -0.132 | -0.065 |
| tCho/tCr | MCI (22) | 0.144 | 0.241 |
|  |  |  |  |
| dPCC | HC (40) | -0.087 | 0.032 |
| mIns/tCr | MCI (22) | -0.243 | -0.332 |
|  |  |  |  |
| dPCC | HC (40) | 0.165 | 0.231 |
| Glx/tCr | MCI (22) | -0.196 | -0.190 |
|  |  |  |  |
| l. HPC | HC (37) | 0.144 | 0.153 |
| tNAA/tCr | MCI (19) | **0.457*** | 0.212 |
|  |  |  |  |
| l. HPC | HC (37) | -0.035 | -0.070 |
| tCho/tCr | MCI (19) | 0.207 | 0.235 |
|  |  |  |  |
| l. HPC | HC (37) | -0.023 | 0.014 |
| mIns/tCr | MCI (19) | 0.123 | -0.122 |
|  |  |  |  |
| l. HPC | HC (37) | 0.250 | 0.146 |
| Glx/tCr | MCI (19) | 0.133 | 0.029 |
|  |  |  |  |
| l. SM1 | HC (41) | 0.284 | 0.193 |
| tNAA/tCr | MCI (23) | 0.248 | 0.168 |
|  |  |  |  |
| l. SM1 | HC (41) | 0.137 | 0.067 |
| tCho/tCr | MCI (23) | -0.042 | 0.032 |
|  |  |  |  |
| l. SM1 | HC (41) | 0.117 | 0.015 |
| mIns/tCr | MCI (23) | 0.039 | -0.194 |
|  |  |  |  |
| l. SM1 | HC (41) | 0.064 | -0.062 |
| Glx/tCr | MCI (23) | 0.032 | -0.157 |
|  |  |  |  |
| r. dlPFC | HC (40) | 0.231 | 0.260 |
| tNAA/tCr | MCI (22) | 0.094 | **0.390†** |
|  |  |  |  |
| r. dlPFC | HC (40) | -0.105 | -0.113 |
| tCho/tCr | MCI (22) | -0.213 | 0.120 |
|  |  |  |  |
| r. dlPFC | HC (40) | 0.009 | -0.060 |
| mIns/tCr | MCI (22) | -0.016 | -0.172 |
|  |  |  |  |
| r. dlPFC | HC (40) | 0.064 | -0.040 |
| Glx/tCr | MCI (22) | -0.042 | -0.115 |
|  |  |  |  |

AP = Anteroposterior, ML = Medio-lateral, Vcop = Centre of pressure mean velocity, dPCC = dorsal posterior cingulate cortex, l. HPC = left hippocampus, l. SM1 = left sensorimotor cortex, r. dlPFC = right dorsolateral prefrontal cortex, tNAA = total N-acetyl aspartate, tCho = total choline, mIns = myoinositol, Glx = glutamate//glutamine complex, tCr = total creatine. * p < 0.05, ** p < 0.01, **†** p < 0.1 with a moderate effect side (|r| > 0.3).

**Figure A.1. Group differences in sway activity**


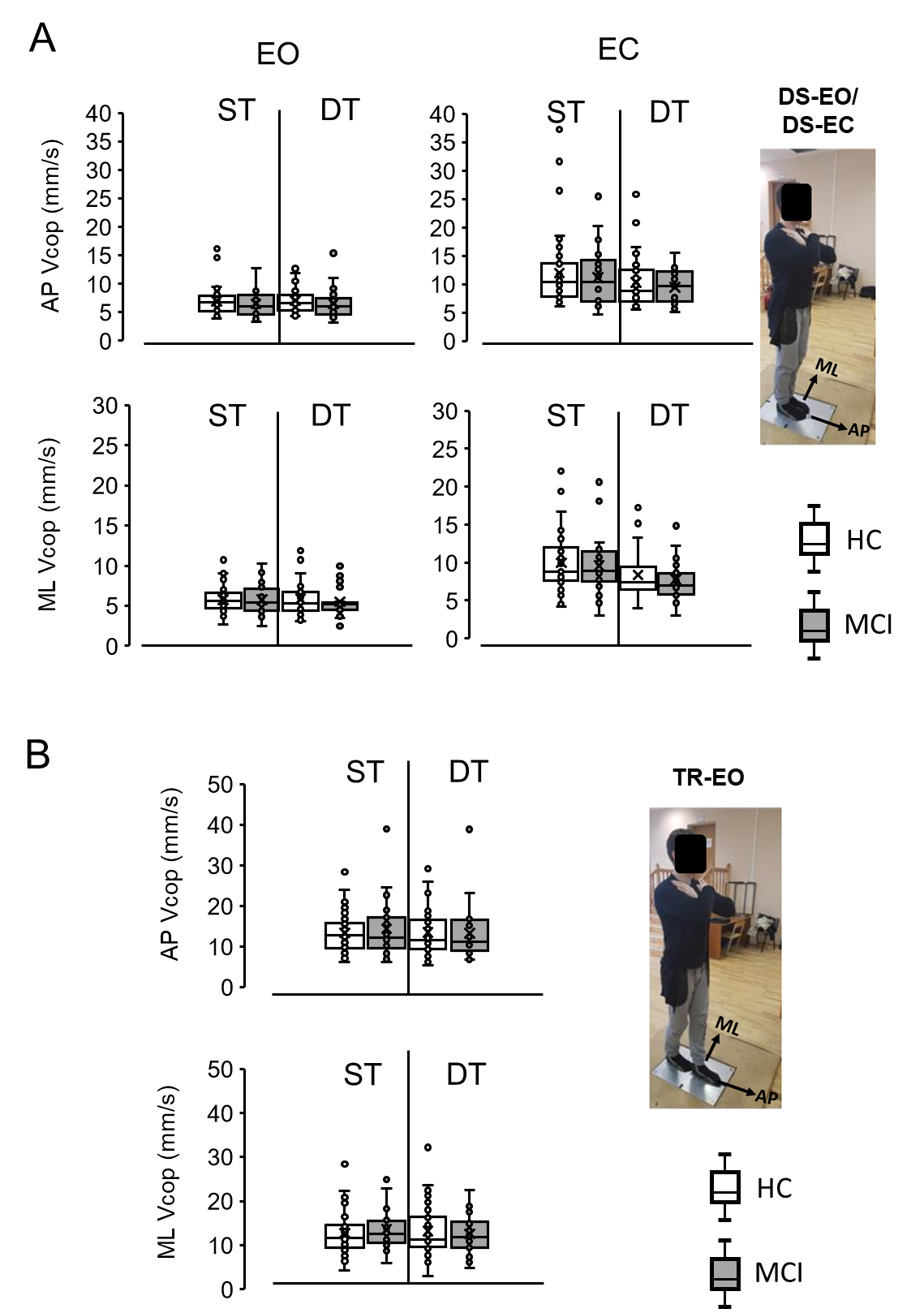


**Figure A.2.** **Group differences in dual task effect**


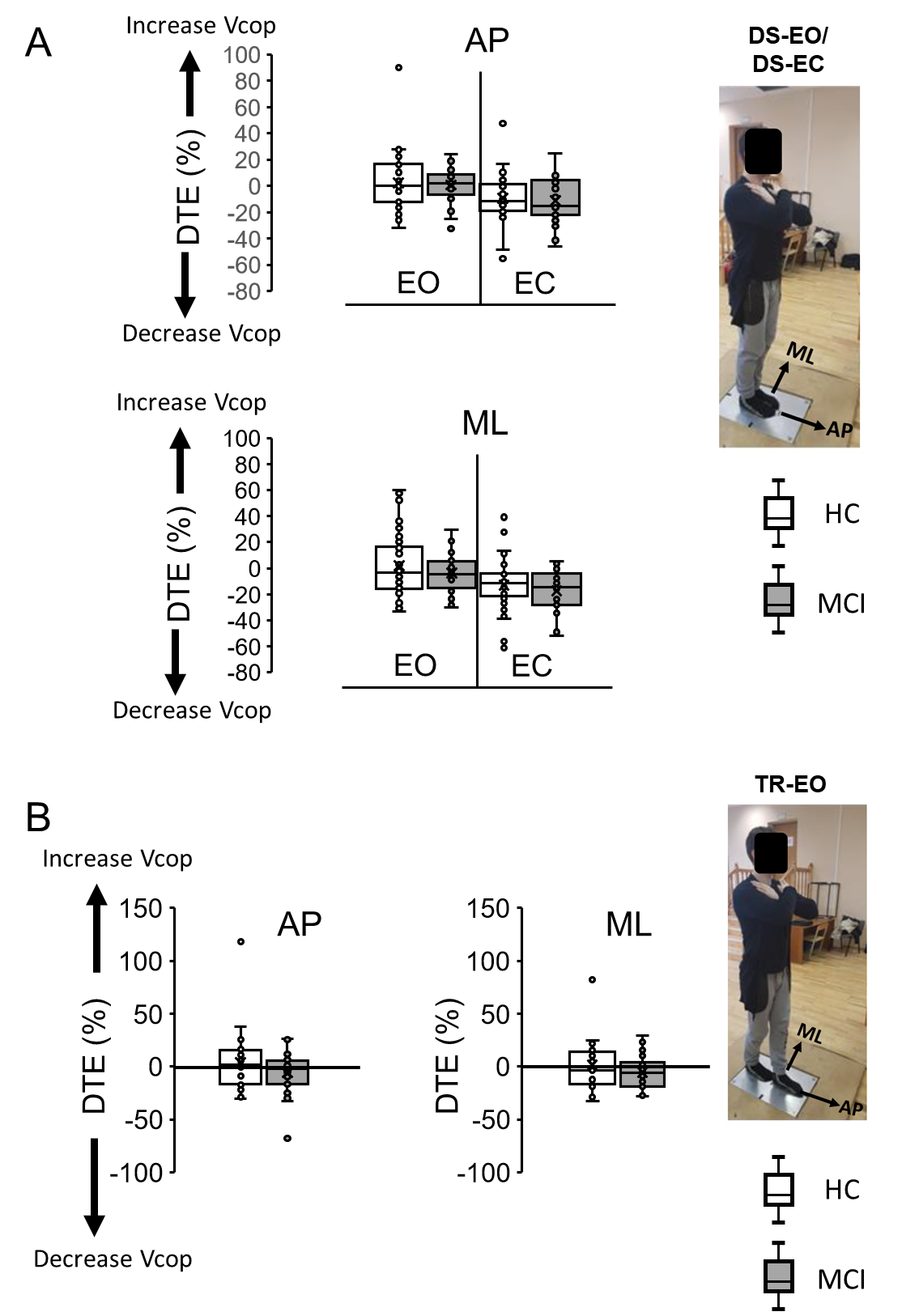


**Figure A.3. Group differences in neurometabolite ratios**

**
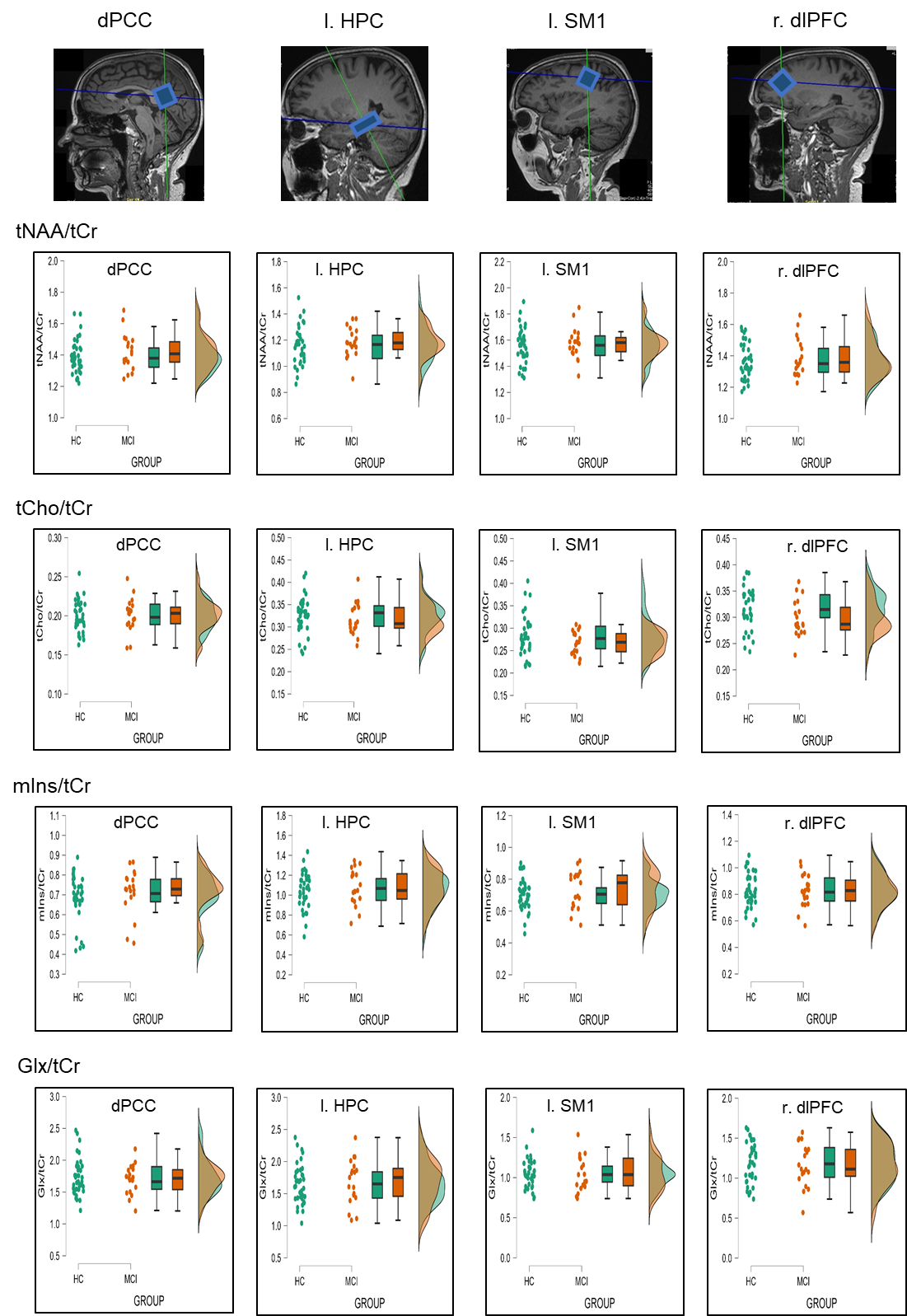
**

**Appendix B:** **MRSinMRS Reporting Checklist for single-voxel MR spectroscopy imaging^1^**

| 1. Hardware 2. Field strength: 3T 3. Manufacture: Siemens 4. Model: Skyra (VE11E) 5. RF coil: 32 channels ^1^H- head coil 6. Acquisition 7. Pulse sequence: PRESS 8. Volume of interest (VOI) locations: 9. dorsal posterior cingulate cortex (dPCC) 10. left primary sensorimotor cortex (left SM1) 11. left hippocampus (left HPC) 12. left medial temporal cortex (left MTC) 13. right dorsolateral prefrontal cortex (right dlPFC) 14. Nominal VOI size: 15. 16 × 16 × 16 mm^3^ (dPCC, left SM1, right dlPFC) 16. 20 × 12 × 16 mm^3^ (left MTC) 17. 26 × 12 × 12 mm^3^ (left HPC) 18. Repetition time (T_R_) and echo time (T_E_) 19. T_R_ = 2000 ms, 20. T_E_ = 30 ms 21. Total number of acquisitions per spectrum: 128 averages 22. Spectral bandwidth: 2000 Hz 23. Number of spectral points: 1024 points 24. Water suppression method: CHESS (bandwidth = 50 Hz) 25. Shimming method: Automated B0-field mapping followed by manual adjustment to reduce water signal FHWM below 15Hz |
| --- |
| 1. Analysis software and outputs 2. Analysis software: LCModel 6.3.1-R 3. Output measures: Ratio to total creatine (tCr) of tNAA, tCho, mIns, and Glx 4. Processing steps: estimation of water-referenced values of total NAA (tNAA = NAA + NAAG), total choline (tCho = GPC + PCh), mIns, and glutamate-glutamine complex (Glx = Glu + Gln) |
| 1. Fitting model basis set   27 basis spectra including: alanine (Ala), aspartate (Asp), creatine (Cr), phosphocreatine (PCr), γ-aminobutyric acid (GABA), glucose (Glc), glutamine (Gln), glutamate (Glu), glycerophosphocholine (GPC), phosphorylcholine (PCh), myo-inositol (mIns), lactate (Lac), N-acetyl aspartate (NAA), N-acetyl-aspartyl-glutamate (NAAG), scyllo-Inositol (Scyllo), taurine (Tau), negative creatine methylene (-CrCH2), guanidinoacetate (Gua), lipids [Lip09, Lip13a, Lip13b, and Lip20] and macromolecules [MM09, MM12, MM14, MM17 and MM20] |
| 1. Data quality 2. Data exclusion criteria: FHWM > 15 Hz, signal to noise ratio (SNR) < 5, or Cramér-Rao lower bound (CRLB) > 20 % 3. Reported measures of SNR and FWHM (in ppm) as reported by LCModel for included spectra:   i. dPCC (64 spectra), SNR [16.6 ± 3.37 (10-27)], FWHM [0.063 ± 0.016 (0.033 – 0.105) ppm]  ii. left SM1(65 spectra), SNR [21.2 ± 4.44 (11-32)], FWHM [0.069 ± 0.016 (0.033 – 0.095) ppm]   1. left HPC (58 spectra), SNR [8.75 ± 1.69 (6-12)], FWHM [0.076 ± 0.014 (0.043 – 0.105) ppm] 2. left MTC (52 spectra), SNR [11.1 ± 2.37 (5-17)], FWHM [0.076 ± 0.014 (0.043 – 0.095) ppm] 3. right dlPFC (64 spectra), SNR [17.9 ± 4.03 (10-26)], FWHM [0.077 ± 0.019 (0.033 – 0.105) ppm] 4. CRLB as reported by LCModel for included spectra:   i. dPCC: tNAA [3.67 ± 0.77 (2-6) %], tCho [4.67 ± 0.69 (3-6) %], mIns [5.39 ± 0.77 (4-9) %], Glx [7.03 ± 1.58 (5-13) %], tCr [3.02 ± 0.49 (3-6) %]  ii. left SM1: tNAA [3.08 ± 0.87 (2-5) %], tCho [3.49 ± 0.56 (2-5) %], mIns [5.34 ± 1.05 (3-9) %], Glx [11.1 ± 2.40 (6-17) %], tCr [2.82 ± 0.56 (2-4) %]  iii. left HPC: tNAA [5.83 ± 1.20 (3-8) %], tCho [4.75 ± 0.96 (3-7) %], mIns [5.31 ± 1.19 (3-9) %], Glx [9.81 ± 2.53 (6-19) %], tCr [4.36 ± 0.74 (3-6) %]   1. left MTC: tNAA [5 ± 1.34 (3-8) %], tCho [4 ± 0.97 (3-7) %], mIns [5.65 ± 1.66 (3-11) %], Glx [8.65 ± 2.17 (5-16) %], tCr [4.85 ± 0.85 (3-6) %] 2. right dlPFC: tNAA [3.30 ± 1.00 (2-6) %], tCho [3.42 ± 0.59 (3-5) %], mIns [4.62 ± 1.03 (3-10) %], Glx [10.5 ± 2.84 (7-18) %], tCr [2.94 ± 0.59 (2-4) %] |

**^1^**In line with the guidelines presented in Lin A, Andronesi O, Bogner W, et al. Minimum Reporting Standards for in vivo Magnetic Resonance Spectroscopy (MRSinMRS): Experts' consensus recommendations. NMR in Biomedicine 2021; 34: e4484.
